# Treatment duration of febrile urinary tract infection (FUTIRST trial): a randomized placebo-controlled multicenter trial comparing short (7 days) antibiotic treatment with conventional treatment (14 days)

**DOI:** 10.1186/1471-2334-9-131

**Published:** 2009-08-19

**Authors:** Cees van Nieuwkoop, Jan W van't Wout, Willem JJ Assendelft, Henk W Elzevier, Eliane MS Leyten, Ted Koster, G Hanke Wattel-Louis, Nathalie M Delfos, Hans C Ablij, Ed J Kuijper, Jan Pander, Jeanet W Blom, Ida C Spelt, Jaap T van Dissel

**Affiliations:** 1 Department of Infectious Diseases, Leiden University Medical Center, Leiden, The Netherlands; 2 Department of Internal Medicine, Bronovo Hospital, The Hague, The Netherlands; 3 Department of Public Health and Primary Care, Leiden University Medical Center, Leiden, The Netherlands; 4 Department of Urology, Leiden University Medical Center, Leiden, The Netherlands; 5 Department of Internal Medicine, Medical Center Haaglanden, The Hague, The Netherlands; 6 Department of Internal Medicine, Groene Hart Hospital, Gouda, The Netherlands; 7 Department of Internal Medicine, Spaarne Hospital, Hoofddorp, The Netherlands; 8 Department of Internal Medicine, Rijnland Hospital, Leiderdorp, The Netherlands; 9 Department of Internal Medicine, Diaconessenhuis Leiden, The Netherlands; 10 Department of Medical Microbiology, Leiden University Medical Center, Leiden, The Netherlands; 11 Department of Clinical Pharmacy and Toxicology, Leiden University Medical Center, Leiden, The Netherlands; 12 Primary Health Care Center, Wassenaar, The Netherlands

## Abstract

**Background:**

Current guidelines on the management of urinary tract infection recommend treating febrile urinary tract infection or acute pyelonephritis with antimicrobials for at least 14 days. Few randomized trials showed the effectiveness of treatment durations of 5 to 7 days but this has only been studied in young previously healthy women.

**Methods/Design:**

A randomized placebo-controlled double-blind multicenter non-inferiority trial in which 400 patients with community acquired febrile urinary tract infection will be randomly allocated to a short treatment arm (7 days of ciprofloxacin or 7 days of empirical β-lactams ± gentamicin intravenously with early switch to oral ciprofloxacin followed by 7 days of blinded placebo) or standard treatment arm (7 days of ciprofloxacin or 7 days of empirical β-lactams ± gentamicin intravenously with early switch to oral ciprofloxacin followed by 7 days of blinded ciprofloxacin). The study is performed in the Leiden region in which one university hospital, 6 general hospitals and 32 primary health care centers are clustered. Patients eligible for randomization are competent patients aged 18 years or above with a presumptive diagnosis of acute pyelonephritis as defined by the combination of fever, one or more symptoms of urinary tract infection and a positive urine nitrate test and/or the presence of leucocyturia. Exclusion criteria are known allergy to fluoroquinolones, female patients who are pregnant or lactating, polycystic kidney disease, permanent renal replacement therapy, kidney transplantation, isolation of ciprofloxacin-resistant causal uropathogen, renal abscess, underlying chronic bacterial prostatitis, metastatic infectious foci and inability to obtain follow-up. The primary endpoint is the clinical cure rate through the 10- to 18-day post-treatment visit. Secondary endpoints are the microbiological cure rate 10- to 18-day post-treatment, the 30- and 90-day overall mortality rate, the clinical cure rate 70- to 84-day post-treatment, relapse rate of UTI and adverse events or complications during 90 days of follow-up.

**Discussion:**

This study aims to demonstrate that 7 days of antimicrobial treatment is non-inferior as compared with 14 days of treatment in patients with febrile urinary tract infection. In addition, it will generate insights into the side-effects of antimicrobial treatment in relation to its duration. The study population will also include men, the elderly and patients with significant co-morbidity. Reflecting daily practice of primary health care and emergency departments, the results of this study can be generalized to other locations.

**Trial registration:**

(Trial registration at clinicaltrials.gov: NCT00809913 and trialregister.nl: NTR01583)

## Background

Fever in urinary tract infection (UTI) suggests the presence of tissue inflammation and is therefore considered to be a sign of acute pyelonephritis (AP) [1,2]. In 2000, the estimated direct and indirect costs of acute AP in the United States were $2.14 billion which is predominantly related to hospitalization [3]. In the last decades hospitalization rates of patients with AP has decreased from almost 100% to 10–30% [4-6]. The outpatient management of patients with AP has become popular as have oral antimicrobial treatment regiments and shortening of treatment duration [7-10]. However, such approaches have only been studied in the setting of uncomplicated AP, defined as AP in a young otherwise healthy non-pregnant woman without co-morbidity. In contrast to this, the optimal treatment of patients with complicated AP remains unclear and, not surprisingly, treatment guidelines therefore almost exclusively discuss uncomplicated AP [11,12]. Current strategies recommend antimicrobial treatment for at least 14 days [13-16].

Facing the aging of the general population it is challenging to better define the optimal treatment for AP in an unselected population including the elderly, men and patients with co-morbidity. Moreover, fluoroquinolones, being the preferred oral empirical treatment for AP, are associated with considerable side effects such as dysglycemia and *Clostridium difficile *infections, especially in the elderly and subjects with co-morbidity [17,18]. Therefore it is questionable whether the benefit of antimicrobial treatment of AP at the end of a 14-day antimicrobial treatment still outweighs its potential side effects.

We will conduct a multicenter, randomized, double-blind, non-inferiority trial to determine whether the efficacy and safety of a 7-day antimicrobial regimen is similar to a 14-day antimicrobial regimen in unselected individuals presenting with AP, defined as febrile UTI, at primary care or emergency department.

## Methods/Design

A randomized placebo-controlled double-blind multicenter trial is conducted with the aim to determine non-inferiority of the primary outcome measure between short treatment and standard treatment of febrile UTI. Consecutive patients will be randomized in a 1:1 ratio stratified per center and sex, to receive either a 7-day or 14-day regimen of antimicrobial treatment. Those enrolled in the 7-day regimen will be provided with placebo tablets to match antimicrobial tablets to complete a 14-day course.

### Patients

#### Inclusion criteria

A subject is eligible for inclusion if all of the following criteria apply: 1) Competent patient aged 18 years or above, 2) One or more solicited symptoms suggestive of urinary tract infection (UTI) (i.e. dysuria, frequency, urgency, perineal pain, suprapubic pain, costovertebral tenderness or flank pain), 3) Fever defined as a measured temperature ≥ 38.2°C (ear, rectal) or ≥ 38.0°C (axillary) and/or a history of feeling feverish with shivering or rigors including the past 24 hours, 4) Positive urine nitrate test and/or leukocyturia as depicted by leukocyte esterase test or the presence of more than 5 leukocyte per high- power field in a centrifuged sediment.

#### Exclusion criteria

A subject is not eligible for inclusion in this trial if any the following criteria apply: 1) Known allergy to fluoroquinolones, 2) Pregnancy or lactation, 3) Polycystic kidney disease, 4) Permanent renal replacement therapy, 5) History of kidney transplantation, 6) Residence outside country of enrollment, 7) Inability to speak or read Dutch and 8) Absence of written informed consent.

In addition to these exclusion criteria patients with the following conditions, indicating longer or other antimicrobial treatment, will not be randomized: 1) Isolation of ciprofloxacin-resistant causal uropathogen, 2) Presence of renal abscess, 3) Presence of underlying chronic bacterial prostatitis and 4) Presence of metastatic infectious foci.

A subject can be included in this study only once.

#### Setting

**Figure 1:**
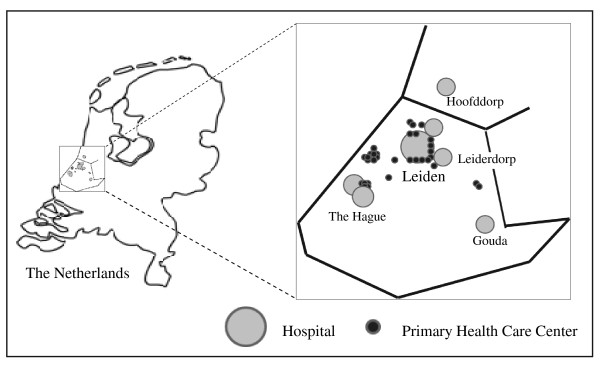
**Geographic distribution of participating sites**.

Patients eligible for this trial will be recruited at primary health care centers or at emergency department (ED) of hospitals in the Leiden region. The primary care region and participating hospitals serve one single area (North-West of South Holland) of the Netherlands (Figure 1). Patients considered ill enough by their primary care physician to require hospitalization will be enrolled at the emergency department of affiliating regional hospitals to which they refer.

### Treatment

Subjects will be randomized to receive either short antimicrobial treatment for 7 days followed by placebo for 7 days, or standard antimicrobial treatment for 14 days. The decision whether to treat as outpatient or inpatient will be made by the attending physician based on clinical judgment. Outpatients will be treated non-blinded with oral ciprofloxacin (500 mg b.i.d.) for 7 days followed by the blinded study medication (oral ciprofloxacin 500 mg or placebo b.i.d.) for 7 days. Inpatients will be empirically treated according to local guidelines on antimicrobial treatment (β-lactam ± aminoglycoside intravenously). After empirical intravenous treatment, patients will be switched as soon as possible to oral ciprofloxacin. Patients will then receive non-blinded oral ciprofloxacin (500 mg b.i.d.) up to the 7^th ^day followed by the blinded study medication (oral ciprofloxacin 500 mg or placebo b.i.d.) for additional 7 days. Inpatients that are empirically treated with oral antimicrobials will be treated as the outpatients. The placebo is indistinguishable from the ciprofloxacin containing formula, by sight and taste. Blinded treatment (ciprofloxacin 500 mg or placebo) is manufactured according to Good Manufacturing Principles principles by ACE Pharmaceuticals BV, Zeewolde, The Netherlands. Both the subject and investigator are blinded.

Patients with an indication of longer or other antimicrobial treatment as assessed by the additional exclusion criteria will be treated according to best clinical practice as judged by the attending physician. Though not randomized, these patients will remain in the study to assess the outcome.

### Data collection and management

Demographic, clinical and microbiological data will be collected by qualified research nurses or the clinical investigator (CvN) who will visit all patients at home directly after they have been notified by the primary care physicians. Data from patients included at the ED will be collected from the medical record completed with an interview by telephone or at the bedside using a standardized questionnaire. Blood and urine cultures will be taken before commencement of antimicrobial therapy and are analyzed by standard microbiological methods at local certified laboratories. All patients will be contacted by person at day of enrolment, 3–4 days and 28–32 days thereafter and are contacted by phone at 13–15 days and 84–92 days after enrolment to assess outcome. Urine cultures will be performed 28–32 days after enrolment to assess microbiological outcome. In case of (re)admission during the study period, related data will be taken from the medical record. A detailed flowchart of data registration is outlined in Table 1.

**Table 1:** Flowchart of assessments in patients with acute pyelonephritis at time points during follow-up.

Evaluation	Days after Enrolment				
	
	0	3–4	13–15	24–32	84–92
**Notification to study center**	X				
**Entry criteria**	X				
**Written Informed Consent**	X*	X*			
**Demography**	X				
**Clinical data**	X	X	X	X	X
**(Serious) Adverse events**	X	X	X	X	X
**Mortality**		X	X	X	X
**Blood culture**	X				
**Urine culture**	X			X	
**Randomization and allocation to study treatment**		X			
**End of treatment assessment**			X		
**Contact – in person**	X	X		X	
**Contact – by phone**			X		X

Ad *: After notification at emergency department written informed will be obtained as soon as possible with a maximum till the 4^th ^day.

Data of clinical and microbiological data are collected in standardized questionnaires, case record files (CRFs), subjects' diaries and laboratory records. The data will be entered into a SPSS database using SPSS Data Entry Builder 4.0. To ensure validity of entrance the data will be inputted twice. All data sets will be initiated with an audit trail. The subjects' diaries are considered supportive for the investigators when filling in the CRFs, i.e. all relevant information will be translated and transferred to the CRFs by the research nurses or the clinical investigator. The data sets will be checked for consistency and plausibility. All ambiguous or implausible data items will be resolved by data queries to the investigators in the field.

### Randomization

A computer-generated randomization list of permuted blocks of 4 made by an appointed pharmacist will be used for treatment allocation. This list contains the numbers 1 to 500 and consists of 125 blocks of 4. The list of randomization numbers and corresponding treatment (placebo or ciprofloxacin) will be saved in an independent database with restricted access by the pharmacist. Stratification will be done per center site (7 hospitals; all primary health care centers are considered as one center) and sex. Each collaborating center will be allocated eight consecutive treatment packages consisting of 2 (one for males and one for females) consecutive blocks of 4, each time when needed. Randomization, and thus allocation to study treatment, will be done once the results of the urine culture become available at the 3^rd ^or 4^th ^day after inclusion.

### Endpoints

#### Primary endpoint

The primary outcome measure is clinical cure rate, defined as being alive with resolution of UTI symptoms and fever, among evaluable patients through the 10- to 18-day post-treatment visit.

#### Secondary endpoints

The secondary outcome measures are bacteriologic cure through the 10- to 18-day post-treatment visit, clinical cure rate through the 70- to 84-day post-treatment, all-cause mortality and adverse event rate determined 10- to 18 days and 70- to 84 days post-treatment, rate of UTI relapse and rate of adverse events during 90 days of follow-up. In addition, the outcome measures will be stratified according to final diagnosis as classified by the Infectious Diseases Society of America and the European Society of Clinical Microbiology and Infectious Diseases [19,20]. This includes analysis of specific subgroups, i.e., men, patients with acute complicated pyelonephritis, patients with any co-morbidity and patients with bacteremic UTI.

#### Definitions

The following definitions will be used. *Clinical cure *is defined as being alive with the absence of fever (temperature < 38.0°C) and the resolution of UTI symptoms (either absence of symptoms or at least 2 points improvement on a 0 through 5 points severity score) through the post-treatment visit whereas no additional antimicrobial therapy for a relapse of UTI is described. *Clinical treatment failure *is the opposite of clinical cure and defined as deterioration, persistent fever and/or symptoms of UTI (less than 2 points improvement on a 0 through 5 points severity score) or relapse of UTI with the same uropathogen for which additional antimicrobial treatment is prescribed. *Clinical recurrence *is defined as any clinical UTI after clinical recovery and the end of treatment period including uncomplicated UTI, complicated UTI and acute pyelonephritis as defined previously [19]. Based on microbiological typing of isolated uropathogen distinction will be made between clinical relapse, being the same uropathogen or clinical reinfection with a different uropathogen. *Pre-treatment significant growth of uropathogens *is defined as ≥ 10^4 ^colony forming units/mL in women (midstream urine) or ≥ 10^3 ^colony-forming units/mL in men (midstream urine) or ≥ 10^3 ^colony-forming units/mL (catheter urine) or ≥ 10^2 ^colony-forming units/mL of midstream urine collected during antibiotic treatment of UTI at study entry [19]. *Bacteriologic cure *or eradication is defined as the elimination of the study entry uropathogen or as pathogen growth of less than 10^4 ^colony forming units/mL in women or less than 10^3 ^colony-forming units/mL in men, of a midstream urine sample collected combined with the disappearance of leucocyturia [19]. *Bacteriologic failure *through the 10- to 18-day post-treatment visit is defined as the absence of bacteriologic cure and classified as persistence of the original pathogen or superinfection with a new pathogen on the basis of species determination and antibiogram or an alternative method, such as Amplified Fragment Length Polymorphism or sequence-based PCR using repetitive extragenic palindromic primers. *Ciprofloxacin resistance *of uropathogens is defined by a MIC > 4 mg/L and includes all *Enterococcus spp. Acute complicated pyelonephritis *is defined as febrile urinary tract infection in a person with one or more of the following baseline characteristics: male sex, postmenopausal (female aged ≥ 50 years), underlying urinary tract disorder (e.g. nephrolithiasis, urologic malignancy, vesicoureteral reflux, urethral stricture), diabetes mellitus, immunocompromised, renal insufficiency or progressive UTI despite antimicrobial UTI treatment. *Co-morbidity *is defined as the presence of any urinary tract disorder, heart failure, cerebrovascular disease, renal insufficiency, diabetes mellitus, malignancy or chronic obstructive pulmonary disease for which the patient is prescribed medication and/or consults a hospital-based medical specialist.

#### Ethics

This study is conducted in accordance with the principles of the Declaration of Helsinki and 'good clinical practice' guidelines. The independent medical ethics committees of the participating centers have approved the study protocol. Written informed consent is obtained prior to randomization; consent by proxy will be obtained for patients who are temporally unable to provide consent.

### Statistical analysis

#### Sample size

**Figure 2:**
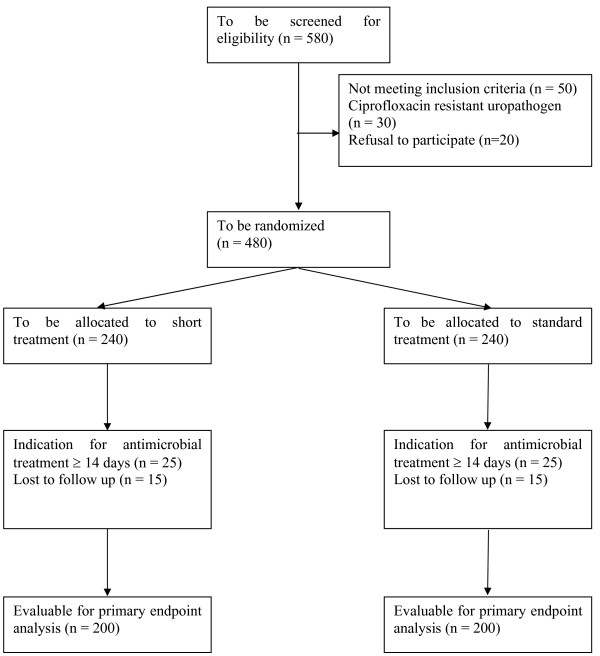
**Recruitment of consecutive patients with acute pyelonephritis**.

From previous studies the clinical cure rate is estimated to be approximately 90% [9,10,21]. This study will have power of 90% to show that the clinical cure rate for Short treatment (7 days followed by placebo for 7 days) is at least as high as the cure rate for Standard treatment (14 days). Assuming that the event rates for the Standard treatment and Short treatment populations are 90% and 89.5%, respectively, that a difference of 10,0 points or less is unimportant, and that alpha (1 tailed) is set at 0.05, the sample size in the two groups will be 200 each. Formally, the null hypothesis is that the clinical cure rate for Short treatment is 10,0 percentage points lower than the cure rate for Standard treatment, and the study has power of 90% to reject this null. Equivalently, the likelihood is 90% that the 95,0% confidence interval (CI) for the difference in clinical cure rates will exclude a 10,0 point difference in favor of Standard treatment. We adopted 10% as the margin of non-inferiority as suggested previously [22]. In order to evaluate 200 patients in both treatment arms and considering subjects who will discontinue the study for various reasons, we estimate that 480 patients are needed to be randomized and 580 patients must be screened for randomization. According to CONSORT the flow of expected participants are outlined in Figure 2[23].

#### Intention to treat, per-protocol and subgroup analysis

The primary endpoint will be analyzed on the Intent-To-Treat population, including all randomized patients who received at least one dose of the study drug, and on the Per-Protocol population, including all randomized patients who had been given the study drug for a minimum of 24 hours (in case of treatment failure) or who had been taken at least 80% of the study drug (in case of clinical cure). The primary significance test will be carried out on the equivalence in cure rates or clinical failure rates in the two treatment arms.

Descriptive statistics will be used to describe the baseline characteristics of the participants in each arm. Binomial or categorical outcome measures will be analyzed using Chi-square tests (Pearson's or Fisher's). Risk difference with 95% CI will be used to compare the differences of categorical outcomes. The primary outcome variable is clinical cure rate assessed 10- to 18 days post-treatment. As we are only interested in non-inferiority and not in equivalence, the sample size calculation is based on a one-tailed alpha of 0.05. This implies that the 90% CI of a two-tailed Chi-square test should not cross the predefined risk difference of 10% lower clinical cure rate [24].

The primary outcome will additionally been tested in the subgroups of males, females, patients with acute complicated pyelonephritis, patients with any co-morbidity, patients aged ≥ 50 years, patients with bacteremic UTI and patients with stepdown treatment (antimicrobials intravenously followed by oral ciprofloxacin during the first 7 days). Subgroup analysis and all secondary outcome measures will be analyzed using Chi-square tests.

In case of significant differences in baseline characteristics, which may have happen by chance as we only stratify randomization by sex and center, the primary outcome will additionally be tested using a multivariable binary logistic regression model to adjust for the following potential confounders: age, acute complicated pyelonephritis, underlying co-morbidity and the use of stepdown treatment.

### Safety

Adverse events will be monitored by passive self-reporting and active surveillance at each follow-up visit or telephone call; the rates of adverse events within the two treatment arms will be compared. Safety data will be subject to clinical review and summarized by appropriate descriptive statistics. In case of treatment failure the attending physician will prescribe additional antimicrobial treatment, as appropriate. Such 'rescue medication' will be offered by the study team.

An independent Data and Safety Monitoring Board (DSMB) will monitor safety on an ongoing basis throughout the trial. Safety reports summarizing Serious Adverse Events (SAE) will be submitted to the Chair of the DSMB after each SAE. In addition, the DSMB will review the results of the interim analyses scheduled to occur after approximately 25, 50 and 75 percent of the patients have completed until the 10- to 18-days post-treatment visit. The objective of the interim analyses is to consider stopping for safety reasons only. The breaking of the blind and the analysis of the data will be performed by a pharmacist and the members of DSMB. The following stopping rules are predefined during each interim analysis: 1). Regarding the primary outcome measure, Short treatment is definite inferior to Standard treatment with statistical significance of ≥ 95% certainty and 2). The clinical cure rate in the Standard arm is <75%. Besides these stopping rules, the judgment of the DSMB will be based upon a general review of adverse events including 30-day mortality. The initial communication to the study team will consist only of the outcome of the decision: either 1) there is sufficient evidence to consider stopping the trial or 2) there is insufficient evidence to consider stopping the trial.

### Registration

This study is registered at clinicaltrials.gov [NCT00809913] and trialregister.nl [NTR01583] with the acronym FUTIRST-trial used.

## Discussion

UTIs in adults are classified into acute uncomplicated UTI, acute uncomplicated pyelonephritis, complicated UTI (CUTI), acute complicated pyelonephritis and in addition, for men there are several categories of prostatitis [11,12,19,25]. The term 'uncomplicated' is usually restricted to non-pregnant women without underlying functional or anatomical abnormalities of the urinary tract but it remains controversial whether older, postmenopausal, women and women with other underlying co-morbidities, like diabetes mellitus, also belong to this group [20]. Pyelonephritis is defined as a serious infection involving the pelvis, calices and kidney parenchyma which is classically characterized by fever, flank pain or costovertebral angle tenderness and signs and symptoms of UTI. While treatment recommendations of acute uncomplicated UTI and acute uncomplicated pyelonephritis are quite straightforward with short oral antimicrobial courses on an outpatient basis, there is a lack of conformity on therapeutic approaches for CUTI and acute complicated pyelonephritis; likely because these conditions reflect a broad spectrum of clinical syndromes. Therefore from a pragmatic viewpoint it is reasonable to classify these patients according to their clinical presentation in which fever reflects whether there is a parenchymal inflammation or not. Whether this involves the kidney, bladder, prostate, lymph nodes of the pelvis, blood circulation or a combination of those cannot be assessed on clinical grounds. In the literature, fever in UTI is mostly attributed to AP [1,2]. In this study we use febrile UTI as the clinical syndrome of interest because this is how patients present and fever mainly determines the appropriate treatment. According to existing classifications this includes CUTI with fever, (un)complicated AP or, as has been suggested, the urosepsis syndrome [26]. However, for reasons of simplicity and clinical pragmatics we consider FUTI as a clinical appearance of AP [1,2].

This study is different from previous studies that address treatment duration of AP. One study included only women with uncomplicated AP and a median age of 25 years [9] while another recent study included predominantly women aged ≤ 45 years of which a small subgroup suffered complicated AP [10]. Based on the results of these studies, it can be concluded that uncomplicated AP can be effectively treated in young women with a 5–7 day course of fluoroquinolones. However, the treatment duration was not the only study objective because the comparators in these studies were other antimicrobial agents. Therefore it remains unclear whether longer treatment of AP with fluoroquinolones might be even better. Other clinical trials on AP did not examine treatment duration but most of them compared newer or extended release fluoroquinolones to conventional ones. In all these studies, treatment duration was variable being 5 to 14 days [27-31]. From these trials it seems evident that AP in many cases can be treated shorter than 14 days. Nevertheless caution is warranted to generalize this to common practice because the study populations were highly selected being predominantly women who were relatively young and healthy. Furthermore, both patients with AP and CUTI were included in which fever was not a prerequisite of inclusion and the majority of cases had CUTI. In other words one might only conclude from these studies that cystitis in a complicated person can effectively be treated with fluoroquinolones less than 14 days. Based on the evidence in the literature we therefore conclude that the duration of antimicrobial treatment in patients with uncomplicated AP could be as short as 5–7 days whereas in patients with complicated AP it is unclear and should be at least 14 days.

In contrast to previous studies, the current study includes consecutive patients with febrile UTI, being AP, reflecting the daily clinical practice of primary care and emergency departments. This includes male participants who by definition are classified as complicated. This might be controversial but from a pathophysiological point of view there is no reason to believe that AP in men behaves different from AP in women. Indeed, previous studies that included a sufficient number of men, showed that treatment of febrile UTI with fluoroquinolones for 7–14 days had comparable clinical cure rates of about 90% [28,30-32]. Moreover, we recently evaluated the guideline of the Dutch College of General Practitioners that recommends antimicrobial treatment of febrile UTI for 10 days irrespective of sex [33]. In this observational study with similar inclusion criteria, male sex was not a risk factor for clinical failure [21]. As in previous studies, the mortality rate in men was higher but this reflects underlying co-morbidity rather than attributable risk of UTI [5,21]. Based on the results of these studies we conclude there is sufficient evidence to include men in such a trial as this. Furthermore, it should be emphasized that although men with suspected chronic bacterial prostatitis are excluded from this trial it might be that men with febrile UTI have indeed acute prostatitis. However, as recommended treatment durations of acute prostatitis are primarily based on expert opinion (being 14 days) and the distinction of febrile UTI in men between pyelonephritis or prostatitis can often not be made clinically, a trial evaluating the optimal treatment duration is even more relevant in this population [34].

In contrast to all previous studies, the study objective of this trial only involves treatment duration. Combined with the inclusion of patients reflecting routine daily practice these aspects are thus unique that it is named with the acronym FUTIRST (Febrile UTI Randomized Short Treatment)-trial to put emphasis on being the first clinical trial on febrile UTI or AP with such a pragmatic design. Meeting the criteria for a practical clinical trial, the results of this study are thus generalizable to other settings and should influence policy makers of treatment guidelines on AP [35].

## Abbreviations

AP: acute pyelonephritis; UTI: urinary tract infection; CUTI: complicated urinary tract infection; SAE: serious adverse event; DSMB: data safety monitoring board.

## Competing interests

The authors declare that they have no competing interests.

## Authors' contributions

CvN and JTvD designed and initiated the study and drafted the protocol. CvN performed the sample size calculation. JWW, WJJA, HWE, EMSL, TK, GHWL, NMD, HCA, EJK, JP, JWB and ICS critically revised the manuscript and locally co-manage the trial and collect data. All authors contributed and approved the final version of the manuscript.

## Pre-publication history

The pre-publication history for this paper can be accessed here:

http://www.biomedcentral.com/1471-2334/9/131/prepub
